# Pediatric Oncologists’ Experiences Returning and Incorporating Genomic Sequencing Results into Cancer Care

**DOI:** 10.3390/jpm11060570

**Published:** 2021-06-18

**Authors:** Rebecca L. Hsu, Amanda M. Gutierrez, Sophie K. Schellhammer, Jill O. Robinson, Sarah Scollon, Richard L. Street, Alyssa N. Salisbury, Stacey Pereira, Sharon E. Plon, Janet Malek, D. Williams Parsons, Amy L. McGuire

**Affiliations:** 1 Center for Medical Ethics and Health Policy, Baylor College of Medicine, Houston, TX 77030, USA; rhsu4@uw.edu (R.L.H.); sks9@rice.edu (S.K.S.); jill.robinson@bcm.edu (J.O.R.); spereira@bcm.edu (S.P.); janet.malek2@bcm.edu (J.M.); 2 Department of Pediatrics, Baylor College of Medicine, Houston, TX 77030, USA; sxscollo@texaschildrens.org (S.S.); splon@bcm.edu (S.E.P.); dwparson@texaschildrens.org (D.W.P.); 3 Texas Children’s Cancer Center, Texas Children’s Hospital, Houston, TX 77030, USA; 4 Department of Communication, Texas A&M University, College Station, TX 77843, USA; r-street@tamu.edu; 5 Department of Medicine, Baylor College of Medicine, Houston, TX 77030, USA; alyssasalisbury@gmail.com; 6 Center for Innovation in Healthcare Quality, Effectiveness & Safety, Michael E. DeBakey Veterans Affairs Medical Center, Houston, TX 77030, USA

**Keywords:** pediatric genomics, return of genomic results, communication, pediatric oncology, exome sequencing, genetic counseling, genomic sequencing, cancer genomics

## Abstract

Pediatric oncologists’ perspectives around returning and incorporating tumor and germline genomic sequencing (GS) results into cancer care are not well-described. To inform optimization of cancer genomics communication, we assessed oncologists’ experiences with return of genomic results (ROR), including their preparation/readiness for ROR, collaboration with genetic counselors (GCs) during ROR, and perceived challenges. The BASIC3 study paired pediatric oncologists with GCs to return results to patients’ families. We thematically analyzed 24 interviews with 12 oncologists at two post-ROR time points. Oncologists found pre-ROR meetings with GCs and geneticists essential to interpreting patients’ reports and communicating results to families. Most oncologists took a collaborative ROR approach where they discussed tumor findings and GCs discussed germline findings. Oncologists perceived many roles for GCs during ROR, including answering families’ questions and describing information in lay language. Challenges identified included conveying uncertain information in accessible language, limits of oncologists’ genetics expertise, and navigating families’ emotional responses. Oncologists emphasized how GCs’ and geneticists’ support was essential to ROR, especially for germline findings. GS can be successfully integrated into cancer care, but to account for the GC shortage, alternative ROR models and access to genetics resources will be needed to better support families and avoid burdening oncologists.

## 1. Introduction

Genomic sequencing (GS) and genomic tests are increasingly being implemented in specialized medical contexts such as pediatric cancer care [1,2]. Integration of GS into the care of children with cancer may play an important role in assessing inherited cancer risks [3], and there is hope among both pediatric oncologists [4] and parents of pediatric cancer patients [5,6] that tumor GS will improve targeted treatment of childhood cancers. However, to return tumor and germline GS results to their patients’ families, oncologists need to take on multiple responsibilities, including interpreting and conveying genomic results and providing emotional support [7]. It is important to develop best practices for integrating genomic testing into pediatric oncology to enhance the patient experience and avoid burdening clinicians [8,9,10].

As genomic testing is integrated into cancer care, interpretation and return of these results is becoming an essential skillset for pediatric oncologists [11,12]. Yet research shows that communication of genomic results can be challenging for non-geneticist physicians [13], especially when conveying information about diseases or conditions unrelated to their area of specialization [14,15,16]. For example, non-geneticist oncologists have reported not feeling confident in their ability to interpret germline information or to effectively communicate non-cancer GS results to families [14,15]. Genetic counselors (GCs) can be a helpful resource when they are available to assist with results disclosure [14]. However, as collaboration between oncologists and GCs to return genomic results together is less common in practice, there are opportunities to explore oncologists’ perspectives around communicating GS information with genetic specialists during the return of genomic results (ROR) process.

We have previously reported pediatric oncologists’ expectations before returning exome sequencing (ES) results to patients’ families [4] and oncologists’ communication through mixed methods analyses of ES ROR sessions [7]. In this manuscript, we evaluate oncologists’ perceptions of the experience of returning tumor and germline ES information to families with GCs to learn how to optimize the implementation of genomic testing into cancer care and to provide additional insight into the practice of ES communication. We describe pediatric oncologists’ experiences communicating ES information to families with support from GCs and genetic specialists, including oncologists’ preparation and readiness for ROR, collaboration with GCs during ROR, and perceived ROR challenges. We discuss the implications these findings have for the incorporation of genomic information into pediatric cancer care.

## 2. Materials and Methods

The BASIC3 (Baylor Advancing Sequencing in Childhood Cancer Care) study was a National Institutes of Health Clinical Sequencing Exploratory Research project conducted between 2012 and 2016 as a collaboration between Baylor College of Medicine and Texas Children’s Hospital (TCH), one of the largest pediatric cancer centers in the U.S. BASIC3 explored the impact of returning clinical germline (blood) and tumor ES results to parents of pediatric cancer patients (under age 18) with solid tumors by oncologists and genetic counselors [3]. Patients with leukemia or lymphoma were not eligible. Parents and pediatric oncologists were enrolled in the study as participants. We approached all TCH pediatric oncologists who were actively treating patients as a part of the solid tumor team, and all oncologists consented to participate in the study. Oncologists attended a 40-min education and consent session before signing a consent form. Further details on the BASIC3 study enrollment, informed consent, and tumor and blood testing have been described elsewhere [3,7,17]. The BASIC3 study was approved by the Institutional Review Board at Baylor College of Medicine.

Patients’ ES results were incorporated into their medical record. Parents received their child’s ES results, significant and non-significant, from their primary pediatric oncologist and a study GC in an ROR session. All patients were offered a disclosure session independent of the type of results found. These sessions were nearly always scheduled on the same day a family was present in the clinic for a regular clinic visit. During the session, all participating families received a paper copy of the patient’s germline sequencing report, and some received a tumor report if a tumor sample was available for sequencing, as well as a letter written by study GCs and a study medical geneticist summarizing the main findings in lay terms. Each session was audio recorded and a communication analysis of the content of these sessions has been previously published [7].

At the time of enrollment, oncologists received education about ES, the design of the BASIC3 study, and the structure of the clinical ES reports (with a review of example reports). Before each oncologist’s first ROR session, they met with two of the study principal investigators (PIs) who are genetic specialists (a medical geneticist and a pediatric oncologist with expertise in molecular precision medicine) and a study GC to review their first patient’s tumor and germline reports and receive an overview of the ROR process. For all subsequent reports, the oncologists were informed of the findings of each report by email and were given the option to request a meeting with the study team to discuss the reports, particularly for significant results. For each case, the primary oncologist and study GCs typically briefly reviewed the report in clinic and discussed a disclosure plan prior to entering the room in order to prepare for results communication to families. These discussions became more abbreviated with increased experience of the oncologist and study GC working as a team but could vary depending on the complexity of the report and disclosure. 

Oncologists were interviewed by an independent investigator after disclosing results to their first five BASIC3 patient families (the “post-disclosure” interview) and then again at least one year later (the “follow-up” interview). The post-disclosure interview guide focused primarily on the oncologist’s experiences during the initial five ROR sessions, how ROR was typically structured, and how the oncologist communicated results. The follow-up interview guide asked questions about the oncologist’s overall experiences in the BASIC3 study (including, but not specifically focused on, ROR), as well as their attitudes towards ES and integrating ES into clinical care (Supplementary File S1).

All semi-structured interviews with oncologists were conducted either in person or over tele/videoconference, audio recorded, and transcribed by a professional transcription company. Interview transcripts were checked alongside recordings for accuracy, analyzed by taking a pragmatic approach to qualitative thematic analysis [18], and managed using the qualitative data analysis program MAXQDA (Version 2018.2.4, VERBI Software GmbH, Berlin, Germany). The interview coding scheme was developed and refined iteratively using both deductive codes to categorize themes in the data based on predefined research questions, and inductive codes to describe themes that arose organically in the data. Three coders (RH, AG, SS) took a consensus coding approach in which each coder first individually identified themes in all transcripts and then together resolved any discrepancies to come to a consensus for all coded text [19]. The coding process incorporated multiple levels of interpretation of the data where coders systematically abstracted content from coded text to assess themes across interviews [18]. The frequencies we report represent the number of unique oncologists whose responses reflected a certain viewpoint. The expression of a viewpoint by some oncologists does not suggest the rest of oncologists expressed the opposite viewpoint. Open-ended interviews allowed for oncologists to express multiple views at once and discuss topics that others may not have found as salient.

## 3. Results

We conducted post-disclosure and follow-up interviews between 2013 and 2017 with 12 pediatric oncologists (24 interviews total) participating in the BASIC3 study. Oncologists had an average of 6 years of practice experience at the time of enrollment in the study (range 0.5 to 20 years). At the end of the study, the 12 oncologists interviewed had each completed an average of 20 ROR sessions (range 8 to 48 sessions). The follow-up interview occurred an average of 15.5 months (range 12 to 33 months) after the post-disclosure interview. Post-disclosure interviews averaged 27 min in length (range 19 to 48 min), while follow-up interviews averaged 22 min (range 17 to 27 min). Here we report thematic findings from both post-disclosure and follow-up time points together and describe themes in pediatric oncologists’ preparation and readiness for returning ES results, collaboration with GCs during ROR, and perceived ROR challenges. Any mention of patients’ “reports” and “results” refer to both tumor and germline reports/results unless otherwise specified.

### 3.1. Oncologists’ Preparation and Readiness for ROR

Most oncologists (*n* = 9) commented that pre-ROR multidisciplinary meetings in which patients’ tumor and germline reports were reviewed with study GCs and genetic specialists were beneficial. These meetings were viewed to be essential to oncologists’ ability to interpret and communicate both tumor and germline ES results to families. Oncologists used the meetings to identify the most relevant findings in the reports and related implications to emphasize for the family, and to assess their readiness in answering family questions about the results. Oncologists described how review meetings were integral to preparing them to deliver information with which they were unfamiliar (Quote 1).

**Quote 1.***“I think that education prior to the disclosure meetings with either Dr. [geneticist PI] or Dr. [oncologist PI] really helps because a lot of the information that comes back on the report is not things that we deal with on a regular basis. I think if we didn’t have those meetings, I think that disclosures would be much more difficult...If there was no review of the reports, it would be a disaster.”* (103, post-disclosure)

Outside of these pre-ROR multidisciplinary meetings, most oncologists (*n* = 8) sought information about patients’ ES results from other sources. Oncologists searched for further information on any major findings, particularly actionable mutations and mutations related to cancer (Quote 2). Oncologists described a need to look up information on findings they were unfamiliar with to prepare to answer families’ questions, but also thought it was “unrealistic” to expect oncologists to be able to explain all identified variants of uncertain significance (VUS) and related genetic syndromes. A few oncologists specifically mentioned that they did not seek more information outside of pre-ROR meetings (*n* = 2), citing a lack of time to do so before each session or noting that the information given in the report and meetings was sufficient to understand the meaning of the mutations.

**Quote 2.***“In the cases where this confirmed a known cancer predisposition syndrome, I’ve turned to other sources, online resources, journal articles, those sorts of things to educate myself more about those syndromes.”* (105, post-disclosure)

Oncologists perceived improvements in their ability to return ES results over time. More than half (*n* = 7) of oncologists described feeling more equipped with practice to interpret ES reports and communicate the results. Oncologists attributed their confidence to the pre-ROR meetings with study GCs and genetic specialists, and felt their comfort in communicating results improved as they participated in more ROR sessions, which allowed them to learn from previous experiences (Quote 3).

**Quote 3.***“I think I’m still evolving and the more information we get, we have been evolving even more. And I think that the last session was going to be with a very anxious family, and the fact that we had more information and more preparation with other experience and other patients actually helped us. I think using this new information will help me in future results disclosures.”* (112, post-disclosure)

### 3.2. Oncologists’ Collaboration with Genetic Counselors during ROR

**Figure 1:**
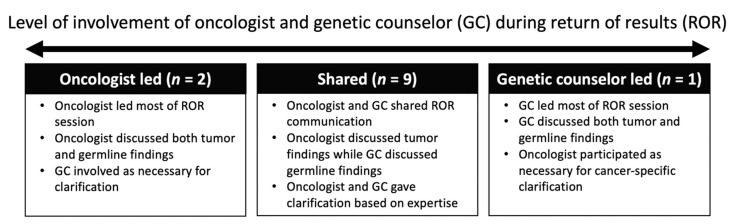
Collaboration between oncologists and genetic counselors during return of results.

Prior to each ROR session, oncologists and GCs discussed how oncologists preferred to be involved in communicating ES results to families during ROR, allowing ROR sessions to be tailored to the oncologists’ preferences for collaboration. In reflecting on the communication structure of their ROR sessions, oncologists described a range of ways in which they worked with the GC (Figure 1).

The majority of oncologists (*n* = 9) described having a shared ROR communication structure with GCs. In this team-based approach, the oncologist discussed the tumor report findings and the GC discussed germline findings, including carrier status, VUS, and relevant non-cancer follow-up testing recommendations for the patient and family (Quote 4).

**Quote 4.***“...Usually, I talk about the [sequencing] of the tumor because that’s where I feel more competent, confident, much easier for me. When we talk about the [sequencing] of the germline...I usually give two sentences saying that ‘No, there is no mutation that may explain why you have tumors, but we found a different type of mutations,’ and the genetic counselor takes over and she explains to them what it really means. To me, that has been a good experience.”* (107, follow-up)

Two oncologists reported leading their ROR sessions, where they communicated the majority of both tumor and germline results to families. These oncologists deferred readily to GCs for additional information or clarification as necessary and to deliver information that the oncologist was less comfortable discussing. Oncologists deferred to GCs most often for germline results, including VUS and carrier status, as well as any implications for cascade testing (Quote 5).

**Quote 5.***“I really focus most of my attention on my patient, then towards the end of the discussion, I will bring the genetic counselor into it if there are implications for testing for the parents, for the family members.”* (105, post-disclosure)

In contrast, one oncologist preferred that the GC lead the ROR sessions, with the GC answering the majority of cancer-specific questions from the family, including how the results impact cancer treatment (Quote 6).

**Quote 6.***“I have to say I tend to take more of a secondary role...I wouldn’t say that I’ve been someone who has been like, ‘Okay, these are the reports, and this is what I’m going to tell you.’ I feel like from one perspective it’s worked better for [the GCs] to do that part and then me to pipe in later, and that may be a comfort level on my end.”* (110, post-disclosure)

Oncologists also differed in their perceptions of their own roles and the GCs’ roles during ROR (Table 1). Oncologists perceived the GCs to have many roles, including helping answer families’ questions (*n* = 4), helping deliver the germline report findings (*n* = 4), and interpreting results in lay language for families (*n* = 2). Two oncologists thought that GCs or genetic specialists should be the ones delivering all ES findings, although that is not necessarily how they structured their own ROR sessions. In terms of their own roles, three oncologists described wanting to rely less on the genetics support team and master ROR themselves, which they saw as part of their professional duty and consistent with the expectations of their patients. Another three oncologists did not think it was their responsibility to gain enough expertise in ES to communicate results to families without support from genetic specialists and saw their role as supporting GCs by discussing the implications of the findings for patients’ cancer management.

### 3.3. Oncologists’ Perceived ROR Communication Challenges

Most oncologists (*n* = 8) discussed communication challenges they experienced during ROR. Six oncologists characterized challenges related to returning uncertain and unknown information to families. Given that the knowledge base of genomics as a field is still emerging, oncologists described how this limited knowledge impacted their own ability to describe both ES results and science to families in lay terms and to convey implications of the findings. They found communication was complicated by having to convey uncertainty around laws governing genetic information and the unknown implications the results could have for patients in the future, including on their ability to obtain insurance outside of the current legislation. In particular, four oncologists described difficulties communicating ES information such as VUS in more accessible language for families (Quote 7).

**Quote 7.***“I think it really tests your skills as a physician and your skills as a communicator. The technology, first of all, is kind of a new one and a challenging one and I think you have to give the family the sense of what was done with the samples...Then you have to sometimes explain results for which there’s a lot of uncertainty. I think some of those categories in the genetic sequencing where we were looking at variants of unknown significance, those were particularly hard.”* (108, follow-up)

Further, oncologists described how the limits of their own expertise in genetics affected their communication (*n* = 5). They described not feeling practiced or trained at interpreting the ES reports without a GC, with one oncologist saying they felt uncomfortable returning potentially “life altering” genetic results to families. Oncologists also voiced not feeling very informed about the standard of care for patients related to genetic information and the immediate implications of the results for the patient (Quote 8).

**Quote 8.***“I can’t say I’m terribly well-informed about some of the mutations. I think the ones that, like this predisposing mutation, [the family is] as well-informed as possible...In that case we specifically mentioned, “I have no idea how it influences the tumor she currently has”...I think they understand that we find a bunch of changes, we don’t necessarily know yet what all those mutations are...I think they get that. But again, I don’t know that I know what to make of that, and I think afterwards they don’t necessarily know what to make of it.”* (114, post-disclosure)

Oncologists also voiced communication challenges related to navigating families’ emotional responses to the results (*n* = 4). They described how the findings (both those with and without clinical significance) unexpectedly triggered family concern, and worried about “piling on bad news” when returning germline results that identified unrelated medical findings. One oncologist cited how giving families more bad news may negatively affect their clinical relationship. They also voiced challenges that arose related to mutations that were shown to be inherited from one parent, which elicited parents’ feelings of guilt and changed the tone of the ROR session (Quote 9).

**Quote 9.***“You can see tension in the room like when you discuss a finding and then you related that that finding came from one parent or the other parent...it always feels a little tense to me. I feel more comfortable when the report shows something that has come from both parents than just one parent...It’s probably just me but I think that you can always kind of see on the parent’s face this kind of feeling of what looks like guilt...One patient recently who had a finding completely unrelated to their cancer, but something related to the rest of their medical history that the mom was the carrier for. I don’t know, I just felt like there was a change in her tone after we discussed it.”* (103, post-disclosure)

## 4. Discussion

This qualitative study explored the experiences of a cohort of pediatric oncologists who participated in a study involving the return of patients’ ES results to their families. We have previously reported that prior to participating in ROR sessions, pediatric oncologists believed that returning ES results would be similar to returning results from other medical tests [4]. However, after participating in ROR, this study found that those same oncologists described feeling they lacked sufficient technical knowledge and training to communicate germline GS results as well as uncertain information in lay terms—similar to challenges identified by non-geneticist physicians in other studies [9,10,13,15,16,20,21,22,23]. As a result, oncologists felt that support from GCs and genetic specialists was essential to interpreting and effectively communicating genomic results to families. These findings are also consistent with other research that showed non-geneticist physicians consulted with outside sources and valued support from GCs and genetic specialists for guidance in returning genomic results to families [9,14,16,20,24,25], especially for germline information [15,20,22]. Our results comparing non-geneticist oncologists’ perceptions of their roles during ROR build upon previous research [26] that showed oncologists viewed their responsibilities related to integrating genomics into their practice to be focused mainly on patients’ cancer management and care.

The results from this study highlight the value of genetics support for non-geneticist physicians during the return of genomic results. Oncologists described positive experiences implementing a collaborative ROR model, which has been used in other GS studies [20,27], where oncologists returned tumor results and GCs returned germline results. However, given the current nationwide shortage of GCs and the increase in non-geneticist physicians returning genomic results, GCs’ efforts in supporting physicians may need to be prioritized for cases in which they can provide their most specialized expertise [14,28,29]. Findings supportive of such an approach have been reported in the adult cancer setting, which has seen the routine integration of GCs into clinical workflow for significantly longer than in pediatrics. Oncologists in these settings have reported the primary utility of genetic testing being the potential impact on clinical decision making, comfort with the ordering of routine genetic tests, and the necessity of recognizing more complex cases requiring a referral for genetic counseling [30]. In some ways, these findings may provide a glimpse into the future of pediatric oncology as the uptake of and familiarity with genetic testing continues to increase.

Non-direct patient care support from genetic specialists may also help fill this gap, as oncologists in this study found benefit in the pre-ROR meetings with specialist physicians (a medical geneticist and oncologist experienced in genomic profiling) to understand the potential implications of ES for patients’ care. Although this type of interaction was not previously codified (or billable) in routine practice, the recent development of electronic consults (“eConsults”) [31] between physicians without the need for a patient visit may support the routine use of this type of interaction in the future. Future research should evaluate such interactions as well as team-based results delivery approaches in pediatric cancer settings where oncologists communicate lower complexity genetic results, such as negative results, but refer more complex cases to GCs or genetic specialists when necessary [29]. These data also support ongoing efforts, including legislative proposals [32], to increase access to genetic counselors and their services equitably to all patient populations when a referral would be of benefit.

These findings have implications for optimizing genomic communication within the parameters of limited physician time and GC supply [8,16,28,33,34]. Future research should build upon existing evidence to examine the efficacy of using different ROR communication configurations in varied clinical cancer contexts and with diverse patient populations. Innovative technological ROR approaches using telegenetics, including chatbots [35], automated disclosure through interactive web-based GS reports [36,37], and bilingual digital results communication platforms [38] have shown promise for communicating genetic results to patients given clinician and resource constraints, and should be evaluated in cancer settings. Research should continue to assess how different genomic communication models among clinicians perform, particularly in telemedicine contexts [27] as telemedicine is already being increasingly used to improve health care access for remote communities [39] and may be leveraged to provide genetic counseling services in settings that lack in-person GCs [40]. In any model assessment, the experiences and satisfaction of GCs as well as of patients and families [41] should be evaluated. In our ongoing research in the Texas KidsCanSeq study [42], we are examining families’ satisfaction and experience with ROR in which GCs are responsible for returning germline results and oncologists separately and individually return tumor findings as a part of regular clinical care. The KidsCanSeq team is also assessing families’ satisfaction with delivering non-significant findings to families in written form through a personalized, detailed letter from GCs and genetic specialists. Notably, the recent passage of the 21st Century Cures Act has resulted in a significant change in the release of test results to patients and parents of pediatric patients as we are also experiencing in our current KidsCanSeq study. Practitioners will need to emphasize pre-disclosure discussions with parents into the types of results they might be expected to see, and development of more just-in-time disclosure methods given the direct availability of results.

Together with previous research [7,9,10,13,14,15,43,44,45,46], our results further emphasize the importance of improving medical education around genetics to train physicians to interpret and communicate genomic information relevant to their practice. In our study, oncologists expressed not feeling practiced or trained at interpreting the genomic results without genetics support. Non-geneticist physicians’ comfort in ROR communication is integral to the future of personalized medicine [15,47], as research shows physicians who had confidence in interpreting genomic results were more likely to use them to plan treatments and discuss them in clinical care [16,25,48]. However, non-geneticist physicians receive limited genetics training in standard medical school curricula or continuing medical education [45,49]. Increased exposure to genetics throughout each level of training to medical specialties [45] could better prepare future physicians to navigate ROR. 

Outside of a medical school setting, genomics communication education and engagement can also be improved for already practicing non-geneticist physicians [45]. Oncologists in this study described feeling their ROR communication improved with time, which is consistent with our previous finding that oncologists used significantly more partnering-supportive speech after their first two ROR sessions [7]. These findings demonstrate the value of ROR practice supported by genetics resources at improving physicians’ confidence in discussing GS results and supporting families during ROR. Others have already outlined genomics communication-specific competencies and training strategies for non-geneticist physicians [50,51,52,53]. Through collaborations between professional medical organizations and central genomics agencies [54,55], standardized print and web-based resources should be developed for non-geneticist physicians to assist with genomic communication [14,56]. Additionally, given their extensive expertise in communicating genomic results, GCs should play a central role in developing these supportive resources and educational materials [57]. In turn, given the increased uptake of genomic somatic testing in the oncology setting, ongoing incorporation and refinement of the curriculum within genetic counseling training programs related to the interpretation of somatic testing should be explored.

Our study had several limitations. First, interviews were completed between 2013 and 2017 and ES technologies have since advanced and may have increased the knowledge base for genomic testing. Next, this study featured retrospective self-evaluation of oncologists’ experiences returning ES results. Also, as the post-disclosure and follow-up interview guides were developed to answer different research questions, we were not able to evaluate how oncologists’ perceptions or comfort may have changed over time. Lastly, our results are from a qualitative interview study with a small sample of pediatric oncologists in a single academic clinical setting that frequently engages with emerging medical technologies and research activities and may not be generalizable to other populations or settings. Future research should examine oncologists’ experiences integrating GS into clinical care in diverse healthcare settings, including community-based clinics that may not be accustomed to research. Of note, our ongoing Texas KidsCanSeq study [42] is currently examining ES in diverse clinical settings and over telemedicine. Despite these limitations, this study provides important insight into oncologists’ experiences returning and incorporating genomic results into cancer care and can inform future work to improve cancer genomics research and practice.

## 5. Conclusions

Despite feeling that their ability to convey genetic information improved over time, pediatric oncologists emphasized how support from GCs and geneticists was essential to returning ES results to their patients’ families. Though there was some variation, most pediatric oncologists preferred to return tumor results while they preferred GCs to return germline results to families. Oncologists also relied on GCs during ROR to communicate ES results in lay terms, discuss implications of the findings, and provide additional support for families. These results are further evidence in support of the integral role GCs play for non-geneticist physicians and their patients. Genomic testing can be successfully integrated into pediatric cancer care. However, to account for the current GC shortage, alternative ROR models and access to genetics resources will be needed to better support families and avoid burdening oncologists.

## Figures and Tables

**Table 1:** Oncologists’ perceptions of genetic counselors’ roles and own roles during return of results sessions.

Themes (Frequency ^1^)	Representative Quote(s)
*Oncologists’ perceptions of GCs’ roles during ROR*	
Help answer families’ questions (*n* = 4)	“I always appreciate having the genetics counselor there because I think the families ask a lot of questions that, just as a medical provider not trained in genetics, you might not be prepared to answer.” (103, post-disclosure) “For tumor report, I think [I’m] well-equipped [to answer questions]. The germline mutations, because I know in advance what the results are and we have these sessions with Dr. [geneticist PI] and genetic counselor, I can estimate how comfortable I will feel answering some questions. So, if I think that it’s not my area of expertise, I just try not to answer the question and say, ‘Here is the person that is better prepared in the field.’” (107, post-disclosure)
Help deliver germline findings (*n* = 4)	“I think having the genetic team with us was exceedingly helpful, probably for two perspectives. One, from a personal perspective of just potentially having someone there as backup. Two, when there were very, very specific targeted questions that parents would occasionally ask, having somebody who really does it all the time there I thought was very good.” (110, follow-up)
Deliver all ES results (*n* = 2)	“I still don’t think I signed up for this stuff...this stuff meaning that that’s what a cancer geneticist does. That’s not my specialty so why am I bearing the brunt of this which I still don’t understand. It’s like this: I tell my colleague who’s a pediatrician, ‘We’re doing this project, and we’re going to see how you deliver the news that your patient has a tumor to a patient.’ He or she didn’t sign up for that, right? That’s not what they’re trained to do...I guess what I’m getting at is why isn’t [a geneticist] delivering this news? Why is it me? That’s their job, right? This is not my job to tell a family that you have a genetic syndrome that predisposed you to cancer.” (104, post-disclosure)
Interpret results into lay language for families (*n* = 2)	“I think [the role of the GC is] mainly the clarifying questions about explaining genes, explaining mutations in a way that’s accessible to the families...At the start of the sessions, I of course touch base with the genetic counselors and ask them to jump in whenever they feel like they need to. So especially if I’m sensing that the way I’m saying something and rephrasing something’s not quite getting the point across, I’ll definitely turn to the genetic counselor.” (105, post-disclosure)
*Oncologists’ perceptions of own roles during ROR*	
Learn to give ES results to their patients (*n* = 3)	“The genetic counselors have been phenomenal and Dr. [geneticist PI] in educating you about these and perhaps I could rely more on them for doing it, but I don’t know, I kind of feel like these are my patients. It’s also something I want to learn to do. I always do these disclosure meetings with the genetic counselors, but I usually lead them and kind of jump in and go for it. I feel like it’s something I should master. I think it’s information they expect me as their physician to be able to relate to them.” (108, post-disclosure)
Support genetic counselor by discussing implications for cancer management (*n* = 3)	“I would say I’m more there as a supporter, to be the one in the room who’s obviously their continuity provider, in a supporter role. [The GC]’s been the one to provide more of the information, which I think is great in this...If I were then to explain how this would change our current management of the patient, I think that would be an appropriate role for me to then be the one to explain that.” (106, post-disclosure)

^1^ Participants’ responses may have fallen under multiple themes.

## Data Availability

Data available from the authors upon request.

## Supplementary Materials

The following are available online at https://www.mdpi.com/article/10.3390/jpm11060570/s1, Supplementary File S1: BASIC3 Study Oncologist Post-Disclosure and Follow-Up Interview Guides.
