# Supplementary material for: Pediatric Oncologists’ Experiences Returning and Incorporating Genomic Sequencing Results into Cancer Care
Source: J Pers Med. 2021 Jun 18;11(6):570. doi: 10.3390/jpm11060570 (PMC8235493; doi:10.3390/jpm11060570)
Supplement: Supplementary file 1 [file jpm-11-00570-s001.zip › jpm-1241754-supplementary.pdf]

Supplementary file S1:
BASIC3 Study Oncologist Post-Disclosure and Follow-Up Interview Guides

Oncologist Post-Disclosure Interview Guide

1. **The last time we spoke, you had not yet returned any tumor or inherited exome results. Tell me what your experience has been like now that you have returned some results to parents.**
    - Describe what the disclosure discussions have been like so far between you and your patients' parents. Could you give me one example?
    - How has your approach to returning the exome results changed over time?
        - What was the first disclosure session like? What have you learned since then?
    - What has come up during the disclosure sessions that you did not anticipate?

2. **When you receive the tumor and inherited exome results, what specific steps do you take to prepare to discuss them with parents?**
    - With whom do you discuss the **tumor** results?
    - What research do you do prior to the **tumor** disclosure meetings with parents?
    - How does your preparation for the **tumor** disclosure vary across patients? On the basis of what?
    - With whom do you discuss the **inherited** results?
    - What research do you do prior to the **inherited** disclosure meetings with parents?
    - How does your preparation for the **inherited** disclosure vary across patients? On the basis of what?

3. **How do you present the tumor and inherited exome results to parents?**
    - Which findings from the **tumor** report do you emphasize?
        - What types of **tumor** results do you tend to focus on?
        - What type of **tumor** results do you tend NOT to focus on?
        - Have there been findings from the **tumor** reports you chose not to discuss at all?
    - Which findings from the **inherited** report do you emphasize?
        - What types of **inherited** results do you tend to focus on?
        - What type of **inherited** results do you tend NOT to focus on?
        - Have there been findings from the **inherited** reports you chose not to discuss at all?
    - Unprompted by you, did any parents say there was some information in the reports that they did NOT want to know?

        [Among parents who asked not to receive some information]
        - What information did they not want to receive?
        - What did you do in this circumstance?
    - How have parents' preferences guided your decisions about what to discuss?

4. **What types of questions have parents asked during the disclosure sessions?**

- What are the most common questions parents have asked? Are there any questions that especially stand out?

For the tumor report:

- Do you feel you have been equipped to answer the questions they've asked?
- Have there been any questions you have not able to respond to?
- What questions should parents have asked that they didn't?
- How have your views on this changed from your first disclosure session to your most recent disclosure session?

For the inherited report:

- Do you feel you have been equipped to answer the questions they've asked?
- Have there been any questions you have not able to respond to?
- What questions should parents have asked that they didn't?
- How have your views on this changed from your first disclosure session to your most recent disclosure session?

5. **Describe your role during the disclosure discussion(s) with parents thus far.**
    - What has been important to you in deciding how to involve **parents** in disclosure discussions?
    - How have you involved your **patients** in the disclosure discussions?
    - How have the roles you just described during discussions of the tumor report been similar to roles during discussions of the inherited report?
        - How have they been different?
    - How have you involved the **genetic counselor** in discussions of the inherited report?

6. **How has the availability of the tumor report impacted the way you are treating your patients' cancers?**
    - How useful has information pertaining to mutations with established clinical utility for the tumor type tested been?
    - How useful has information pertaining to mutations with potential clinical utility (includes genes that are members of cancer pathways, gene families, or functional groups that are targets of approved or investigation therapeutic agents) been?
    - How useful has information pertaining to mutations in consensus cancer genes not included in the first two categories been?
    - How useful has information pertaining to all other mutations been?
    - What challenges has the availability of the tumor mutation information presented in your treatment of patients?
    - How have you utilized the information you obtained from the tumor reports to match your patients to clinical trials/make recommendations about clinical trails at TXCH or at other institutions?
    - Describe a situation when the tumor exome information would be useful beyond your ability to treat your patients.
    - On a scale of 1 to 10, with 10 being most useful and 1 being least useful, how useful has the data from the tumor report been to you?
        - Please tell me how you came up with that number.

7. **How has the availability of the <u>inherited</u> report impacted the way you are caring for your patients?**
    - o How useful has information pertaining to deleterious mutations in disease genes related to cancer susceptibility been?
    - o How useful has information pertaining to variants of unknown clinical significance in genes related to clinical phenotype been?
    - o How useful has information pertaining to immediately actionable mutations been?
    - o How useful has information pertaining to pharmacogentic loci been?
    - o How useful has information pertaining to mitochondrial disorders been?
    - o What challenges has the availability of the inherited mutation information presented in your care of patients?
    - o What recommendations did you make regarding further evaluation for your patients?
        - ▪ For cancer?
        - ▪ For other diseases?
    - o Describe a situation when the inherited exome information would be useful beyond your ability to treat your patients.
    - o One a scale of 1 to 10, with 10 being most useful and 1 being least useful, how useful has the data from the inherited report been to you?
        - ▪ Please tell me how you came up with that number.

8. **What recommendations have you made regarding further evaluation for your patients' <u>parents</u>?**
    - o How have you decided what recommendations to make?
    - o What information from the exome reports has been most useful to you in making these decisions?

9. **What recommendations have you made regarding further evaluation for your patients' <u>siblings</u>?**
    - o How have you decided what recommendations to make?
    - o What information from the exome reports has been most useful to you in making these decisions?

10. **How well informed do you think parents have been after receiving the tumor and inherited reports?**

11. **What impact do you think the tumor and inherited results have had on your patients' parents?**
    - o How have parents reacted to the information in the **tumor** reports?
    - o How have parents reacted to the information in the **inherited** reports?
    - o How do you think their knowing this information has been **beneficial**?
    - o How do you think their knowing this information has been **detrimental**?

12. **Describe your experience with returning <u>carrier status</u> information to parents who opted to receive it.**
    - o How have your patients' parents reacted to the **carrier status** information?
    - o How do you think knowing carrier status information is **beneficial** to parents?

- How do you think knowing carrier status information is **detrimental** to parents?

13. **What impact has the exome info had on your relationships with your patients, your patients' parents and with your patients' siblings?**

14. **How do you feel now about incorporating the tumor and inherited exome results into your patients' electronic medical records?**
    - What are the benefits of this? What are the risks?

15. **Have you/will you order tumor and inherited exome tests for your patients outside of this study as part of their clinical care?**

16. **Now that you have experience returning the tumor and inherited mutation reports to parents, what are your general observations?**
    - What has worked?
    - What has not worked?
    - Describe the most memorable disclosure session you have had.

Oncologist Follow-Up Interview Guide

1. **Let's look back on your experience with returning BASIC3 results. Please describe your overall experience for me.**

- How did you use BASIC3 results?
- If used: Ask about particular cases (one is fine).
  - What was important to you in using results?
- How might WES results be used in the future management of solid organ and brain tumors?
  - What do you think will be important in such future use?

2a. **Let's look back on your impression of the parents' experience with receiving BASIC3 results.**

- Please describe for me what you think the experience of parents with BASIC3 has been like?
- How have parents used the results?
  - Ask about particular cases (one is fine)
- What do you think has been important to parents about WES results?

2b. **Let's look back on your impression of your patients' experience with receiving BASIC3 results.**

- Please describe for me what you think the experience of patients with BASIC3 has been like?
- How have patients used the results?
  - Ask about particular cases (one is fine)
- What do you think has been important to patients about WES results?

3. **How do you think that WES results should be incorporated into the routine management of solid organ and brain tumors in children?**

- If no, please explain your reasons.
- If yes, please tell me some of your reasons.

4. **How do you think that WES might impact the way you practice medicine, now and in the future?**

5. **How do you see WES affecting the practice of pediatric oncology? Pediatrics generally?**

- How do you think WES might contribute to pediatric oncology? Pediatrics generally?

6. **As WES is integrated into care of children with cancers, for what challenges should pediatric oncologists prepare themselves?**

- As WES is integrated into care of children with cancers, for what challenges should parents of patients prepare themselves?
- As WES is integrated into care of children with cancers, for what challenges should pediatric patients prepare themselves?

7. **As WES is integrated into other pediatric subspecialties, for what challenges should your colleagues in those specialties prepare themselves?**

- As WES is integrated into other pediatric subspecialties, for what challenges should parents of patients in those specialties prepare themselves?
- As WES is integrated into other pediatric subspecialties, for what challenges should the patients in those specialties prepare themselves?

8. **Is there anything else about BASIC3 or WES that I have not asked you that you think is important for me to know?**
